# Identification of a new fish trypanosome from the large yellow croaker (*Larimichthys crocea*) and description of its impact on host pathology, blood biochemical parameters and immune responses

**DOI:** 10.1051/parasite/2024078

**Published:** 2025-01-22

**Authors:** Xiaoao Yang, Pengzhi Qi, Zhen Tao, Qingwei Zhang, Yanjie Wang, Denghui Zhu, Xiaojun Yan, Peipei Fu, Baoying Guo

**Affiliations:** 1 National Engineering Research Center of Marine Facilities Aquaculture, Marine Science and Technology College, Zhejiang Ocean University Zhoushan Zhejiang 316022 PR China; 2 School of Fisheries, Zhejiang Ocean University Zhoushan Zhejiang 316022 PR China; 3 Taizhou Key Laboratory of Biomedicine and Advanced Dosage Forms, School of Life Sciences, Taizhou University Zhejiang Taizhou 318000 PR China

**Keywords:** *Trypanosoma*, *Larimichthys crocea*, Identification, Pathology, Immunity

## Abstract

The aim of this study was to clarify the taxonomic identification of a hemoflagellate and assess the effect of trypanosome infection on *Larimichthys crocea*. Giemsa staining showed the presence of three morphotypes of trypomastigotes. The trypanosomes had the following morphological characteristics: a slender body with a long flagellum at the front; body size 12.30–30.90 × 1.13–2.33 μm; elongated oval nucleus situated in the median region; kinetoplast small, oval, located at the posterior end. The parasite had significant morphological differences from *Trypanosoma epinepheli* Su, Feng, Jiang, Guo, Liu & Xu, 2014 and *Trypanosoma carassii* (Mitrofanov) Doflein, 1901. The 18S rDNA sequences of the trypanosome from *L. crocea* had the highest homology (98.4%) with *T. carassii*. Phylogenetic analysis indicated that the parasite clustered with freshwater fish trypanosomes. Based on the differences in morphological characteristics and molecular data, it is considered a new species, *Trypanosoma larimichthysi* n. sp. Trypanosome infection had no effect on the growth of *L. crocea*, but significantly increased the concentration of blood urea nitrogen (BUN), and induced pathological changes in the gills, liver, spleen and kidney. The pro-inflammatory immune genes, including *TNF-α*, *IFN-γ*, *IL-1β*, *CXCL8* and *iNOS*, were significantly upregulated in the *L. crocea* infected with trypanosomes. These results suggest that the trypanosome has negative impacts on host health.

## Abstract (fr)

Le but de cette étude était de clarifier l’identification taxonomique d’un hémoflagellé et d’évaluer l’effet de l’infection trypanosomienne sur *Larimichthys crocea*. La coloration au Giemsa a montré la présence de trois morphotypes de trypomastigotes. Les trypanosomes avaient les caractéristiques morphologiques suivantes : un corps élancé avec long flagelle à l’avant; taille du corps 12,30–30,90 × 1,13–2,33 μm; noyau ovale allongé situé dans la région médiane; kinétoplaste petit, ovale, situé à l’extrémité postérieure. Le parasite présentait des différences morphologiques significatives par rapport à *Trypanosoma epinepheli* Su, Feng, Jiang, Guo, Liu & Xu, 2014 et *Trypanosoma carassii* (Mitrofanov) Doflein, 1901. Les séquences d’ADNr 18S du trypanosome de *L. crocea* présentaient la plus forte homologie (98,4 %) avec *T. carassii*. L’analyse phylogénétique a indiqué que le parasite se regroupait avec les trypanosomes de poissons d’eau douce. Sur la base des différences dans les caractéristiques morphologiques et les données moléculaires, il est considéré comme une nouvelle espèce, *Trypanosoma larimichthysi* n. sp. L’infection par les trypanosomes n’a eu aucun effet sur la croissance de *L. crocea*, mais a augmenté de manière significative la concentration d’azote uréique du sang (BUN) et a induit des modifications pathologiques des branchies, du foie, de la rate et des reins. Les gènes immunitaires pro-inflammatoires, dont *TNF-α*, *IFN-γ*, *IL-1β*, *CXCL8* et *iNOS*, ont été significativement régulés à la hausse chez les *L. crocea* infectés par les trypanosomes. Ces résultats suggèrent que le trypanosome a des impacts négatifs sur la santé de l’hôte.

## Introduction

Fish, an important source of high-quality protein for humans, is more affordable and accessible than other animal-source foods. Aaquaculture has therefore become a vital guarantee ensuring a stable supply of protein for human consumption [10]. In China, fish production from aquaculture has reached 29 million tons, accounting for 52.2% of the country’s total aquaculture output [23]. The rapid expansion of aquaculture over the past few decades has led to the emergence of large-scale and intensive farming models, which are often prone to outbreaks of diseases [25]. In particular, parasitic diseases are becoming increasingly prominent [1, 2, 32], posing significant constraints on the sustainable development of the aquaculture industry.

Trypanosomiasis is a disease caused by *Trypanosoma*, an unicellular flagellated protozoan typically found in the peripheral blood. Since the initial discovery of a piscine trypanosome in the blood of *Salmo fario* in 1841, over 200 species of trypanosomes have been recorded from freshwater and marine fish based on the taxonomic criteria of morphology, geographical and host origin [15]. In China, more than 30 species of trypanosome have been reported in freshwater fish [13], with two species detected in marine fish [5, 25, 40]. Trypanosomes have a wide distribution, including Africa [11, 18, 38], South America [8, 21, 22, 31, 37], Europe [28] and Asia [25, 36, 40], and they are highly prevalent in various fishes, including teleost and elasmobranchs [35].

Traditional taxonomy within the *Trypanosoma* relied on two criteria: morphological characteristics and host specificity. Numerous trypanosomes species have been described based on the “one host – one parasite” paradigm [12]. However, following a series of cross-infection experiments, the concept of strict host specificity for fish parasites was refuted, indicating that the number of species of these flagellates may have been greatly overestimated [46]. The polymorphism exhibited by trypanosomes makes it unreliable to determine new species based solely on morphological differences of the trypomastigote forms [31]. Molecular taxonomy offers a potential solution to this issue. The 18S rRNA gene is the most frequently used molecular marker for the identification and phylogenetic inference of piscine trypanosomes [12]. To date, only eight species of fish trypanosomes have been characterized molecularly, including three species of freshwater trypanosomes and five marine fish trypanosomes [49].

Trypanosomes can elicit a range of clinical symptoms in infected hosts, including anorexia, dorsal depigmentation, anemia and splenomegaly, which can lead to mortality of the hosts [39]. Infection with this hemoparasite is often accompanied by significant alterations in blood parameters [29], diverse levels of organ impairment [7] and a systemic immune response [33, 45]. Due to the unicellular characteristics of trypanosomes, which typically range in size from 10 to 100 μm and can only be observed under a minimum of 400 × magnification, coupled with their unique parasitic site, piscine trypanosomiasis is often difficult to diagnose accurately. It may even be misdiagnosed or missed [42]. Therefore, it has not received adequate attention in aquaculture, and understanding of this disease is relatively limited [38, 42]. However, there has been increased focus on trypanosomiasis recently, particularly in intensive aquaculture. Outbreaks of trypanosomiasis have been reported in net-cage-cultured *Micropterus salmoides* [19], groupers [40, 43], *Lates calcarifer* [25] and blood parrot cichlids [51] in China, as well as Nile tilapia [7, 16] in Sudan and Brazil.

The large yellow croaker is the most productive marine fish species with economically important value in China, and its production reached 258,000 tons in 2022 [23]. However, diseases outbreaks have caused huge losses to the large yellow croaker industry [41]. Recently, we received case reports from local technicians regarding a case of trypanosomiasis in large yellow croaker (*Larimichthys crocea*). Therefore, a detailed investigation into the morphological and molecular characterization of a novel fish trypanosome was conducted, as well as its impact on the pathology, blood biochemical parameters and immune response of large yellow croaker. This study will help increase our understanding of the biology of trypanosomes in large yellow croaker.

## Materials and methods

### Ethics

All animals and studies were conducted in accordance with the rules for the use and care of animals of the Department of Science and Technology of Zhejiang Province “Management of Laboratory Animals in Zhejiang Province”. The Zhejiang Ocean University’s Animal Care and Use Ethics Committee in Zhoushan, China, approved all experimental protocols.

### Farming conditions and sample collection

The study was carried out during a mortality outbreak of larger yellow croaker in Sanduao Bay, Fujian Province, China, induced by trypanosomiasis. Fry of large yellow croaker were provided by Ningde Shengsheng Fishery Technology Co., Ltd, and were cultured in an intensive system (net cage: length 23 m × width 23 m × depth 8 m, with a density of 2,300 fishes/m^3^). Salinity, water temperature, dissolved oxygen, and pH during the disease outbreak were 31.3 ± 1.15 ppt, 20.9 ± 0.46 °C, 5.1 ± 0.58 mg/L and 8.1 ± 0.02, respectively. Large yellow croakers were fed with commercial compound feed once a day at 4 pm.

We randomly collected the large yellow croaker samples from nets in November 2023, including those exhibiting clinical signs of trypanosomiasis and others from distant, unaffected areas showing no symptoms. Samples that tested positive for trypanosome in both blood smear and PCR detection were designated as the infected group, whereas samples that tested negative in both were categorized as the control group (Supplementary file 1). The fish samples were anesthetized with eugenol (0.2 mL/L). The total length (L, cm) and weight (W, g) of each individual were measured to calculate the condition factor (CF = W/L^3^ × 100). After blood drawing and dissection, the tissues of gill, liver, kidney, spleen, heart, and brain were collected for subsequent processing and analysis.

### Blood smear preparation and morphological measurements

Blood smears were prepared by dripping a drop of whole blood onto slides. After air drying, the slides were fixed in absolute methanol and stained with phosphate-buffered Giemsa (Shanghai Yuanye Bio-Technology Co., Ltd, Shanghai, China). The images of trypanosomes were photographed under optical microscopy (ZEISS, Jena, Germany).

Thirteen morphometric parameters were used to describe the morphology of the parasites, including body length (BL), total length with flagellum (TL), free flagellum length (FF), nucleus length (NL), body width (BW), nucleus width (NW), nucleus to anterior end (NA), posterior end to nucleus (PN), kinetoplast to nucleus (KN), posterior end to kinetoplast (PK), the nuclear index (NI = PN/NA, position of nucleus in the body), the kinetoplast index (KI = PN/KN, position of kinetoplast in the body) and flagellar index (FI = FF/BL, position of free flagellum in the body). All measurements were conducted in software Image J (National Institutes of Health, USA), and the units of measurements were in micrometers (μm) unless otherwise stated.

### DNA extraction, PCR amplification and sequencing

Blood was drawn from the caudal vein with a 1 mL syringe. Samples were centrifuged at 3000 r/min for 10 min at 4 °C. Genomic DNA was extracted from the pellet after centrifugation of whole blood using a TIANamp Genomic DNA Kit (TIANGEN, Beijing, China), following the manufacturer’s protocols. The specific primers B (5′-CGAACAACTGCCCTATCAGC-3′) and I (5′-GACTACAATGGTCT CTAATC-3′) were used to amplify the partial fragments of the 18S rDNA gene of trypanosomes [17]. The final volume of the polymerase chain reaction (PCR) was 20 μL, comprising 10 μL of 2xEs Taq Master Mix (Beyotime, Shanghai, China), 10 pmol of each PCR primer, and 1 μL of the extracted DNA templates (>50 ng/μL). The progress of PCR amplification was performed as follows: initial denaturation at 94 °C for 5 min, then 35 cycles of 30 s at 94 °C, 1 min at 56 °C, 1 min at 72 °C, and a final extension of 10 min at 72 °C. The PCR products were sent to Sangon Biotech (Shanghai, China) for sequencing using the PCR primers described above. The obtained sequences were manually assembled with the software SeqMan (DNASTAR, USA), and homology analysis was performed using the blast program available on the NCBI.

### Phylogenetic analysis

Construction of the Bayesian inference (BI) phylogenetic tree was conducted in PhyloSuite [47]. The details were as follows: firstly, the 12 newly sequenced 18S rDNA sequences and 46 published trypanosome 18S rDNA sequences downloaded from GenBank were aligned using MEFFT; then, K2P + I + G4 was selected as the optimal nucleotide substitution model using the BIC implemented in the ModelFinder program; finally, BI phylogenetic analysis was performed using MrBayes with default settings, and 2,000,000 metropolis-coupled MCMC generations. Bayesian posterior probability values were calculated in a consensus tree, after discarding the initial 25% of samples as “burn-in”. MEGA 7.0 Software was used to construct the maximum likelihood (ML) tree with 1,000 bootstraps [20].

### Serum biochemical index detection

The upper serum obtained from blood centrifugation was used for the measurement of biochemical indices. The activity of alanine aminotransferase (ALT) and aspartate aminotransferase (AST), as well as urea (UREA) and creatinine (CREA), the concentration of total protein (TP) and albumin (ALB), were quantified by a biochemical analyzer, using commercially available reagent kits (Seville Biotech Co., Ltd., Wuhan, China). ALT, AST, UREA, CREA, TP and ALB were measured by the Alanine Substrate Method, Aspartic Acid Substrate Method, Urease-GLDH Method, Enzymatic Method, Biuret Method and Bromocresol green, respectively [9, 30].

### Histopathology

The fresh gill, live, spleen, kidney, brain and heart tissues were fixed in a 4% paraformaldehyde (PFA) solution for 48 h. The fixed tissues were dehydrated with aqueous ethanol through an ascending series of concentrations, embedded in paraffin wax, and sectioned at a thickness of 6 μm. The sections were stained with hematoxylin and eosin (H&E) for pathological observation under a light-microscope (Zeiss).

### qPCR

Fresh liver and kidney tissues were kept in RNAlater (Beyotime, Shanghai, China) solution until RNA extraction. Total RNA was extracted using the RNAiso Plus reagent (Takara, Kyoto, Japan). The first-strand of cDNAs was synthesized by a One-Step gDNA Remover kit (Servicebio, Wuhan, China). qPCR was carried out using TB Green^®^ Premix Ex Taq^TM^ (TakaRa) on a CFX96™ real-time detection system (Bio-Rad, Hercules, CA, USA), with the *β-actin* gene serving as the reference gene. The specific primer pairs used in this experiment are detailed in Supplementary file 2. The qPCR cycle conditions were as follows: 95 °C for 30 s, 40 cycles of 95 °C for 5 s, and 60 °C for 34 s, followed by a Melt Curve analysis. The relative expression levels of the target genes were calculated using the comparative Ct method (2^−ΔΔCT^) [24].

### Statistical analysis

Statistical analyses were performed in IBM SPSS Statistics 20 software using student’s *t-*test. Statistical significance was set at *p* < 0.05.

## Results

### Gross observation and prevalence

**Figure 1:**
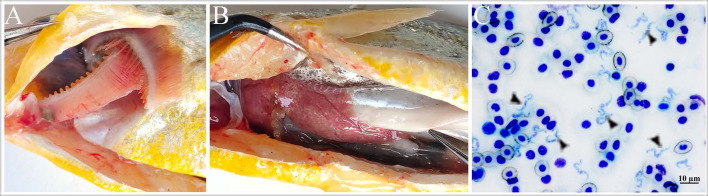
Clinical signs of diseased large yellow croaker. A: Gills of diseased fish. B: Small white nodules densely distributed on the liver. C: Giemsa staining of blood smear in infected fish, and black triangle representing trypanosome, scar bar = 10 μm.

The diseased fish showed typical clinical symptoms of piscine trypanosomiasis [25], including swimming along the pond edges, gasping at the surface of the water, slight darkening of body coloration, and decreased appetite. Gill anemia (Fig. 1A), small nodules on the liver (Fig. 1B) and greyish spleen were usually observed in the diseased fish after necropsy. No leeches were found in the diseased fish. Microscopic examination of blood smears stained with Giemsa indicated that 12 of the 21 sampled fish (57.1%) were infected with hemoflagellates. Trypomastigotes were observed in the blood smears of infected fish (Fig. 1C), with an intensity of 6–70 trypanosomes/100 red blood cells.

### Morphological description and morphometric analysis of trypanosomes

Family Trypanosomatidae Doflein, 1911

Genus *Trypanosoma* Gruby, 1843

#### *Trypanosoma larimichthysi* n. sp.

urn:lsid:zoobank.org:act:2935F644-C3E9-407A-AF26-3985BFE2704A

Type host: *Larimichthys crocea* (Sciaenidae, *Larimichthys*).

Type locality: Ningde, Fujian Province, China (119°31′E, 26°39′N).

Site of infection: Peripheral blood.

Etymology: The specific name is derived from the generic name of the host species.

Type specimens: hapantotypes (ZJOU-LC 202301), parahapantotypes (ZJOU-LC 202306) and voucher specimens (ZJOU-R-2023001B-2023012B) were deposited in the Marine Biology Museum of Zhejiang Ocean University, Zhoushan (Accession no. ZJOU-MBM20241012-1A).

Representative DNA sequences: The newly generated sequences were submitted to the GenBank database under the accession numbers (PP897248–PP897259).

#### Description (Fig. 2, Table 1, *n* = 50)

**Figure 2:**
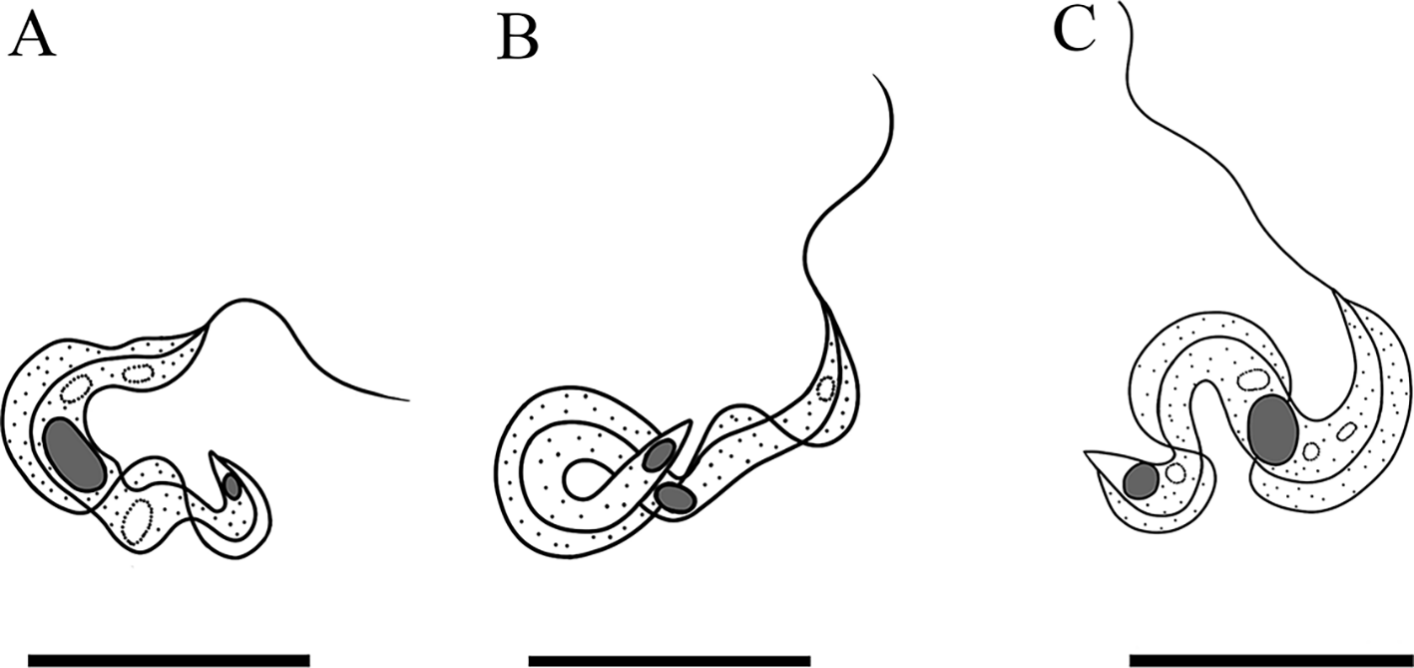
Drawing of Giemsa-staining of three morphotypes of trypanosomes from the blood of *L. crocea*. A: C-shaped; B: yoke-shaped; C: S-shaped.

Three morphotypes of trypomastigotes (C-shaped: Fig. 2A, yoke-shaped: Fig. 2B and S-shaped: Fig. 2C) were found in large yellow croaker. The body is slender body with a long flagellum; the undulating membrane is narrow and distinct (Fig. 2). The flagellum is 8.20–20.87 (15.73) long, accounting for over half of the body length (FI: 0.69). The body length is 12.30–30.90 (23.36), and the width is 1.13–2.33 (1.80). The nucleus is elongated oval, situated in the median region of the body (NI: 1.07); and it is 1.04–2.95 (1.61) long and 0.48–1.41 (0.90) wide. The kinetoplast is small, oval, and located at the posterior end of the body (KI: 1.10) (Fig. 2).

**Table 1:** Measurements of trypomastigotes of *T. larimichthysi* n. sp. in larger yellow croaker from Sanduao Bay in Fujian, China and morphological comparison with described marine and freshwater trypanosomes.

Parameters	*T. larimichthysi* n. sp.	*T. epinepheli*	*T. carassii*
Reference	Present study	Su et al., 2014	Woo., 1981
PK (μm)	0.99 ± 0.26 (0.53–1.60)	5.4 ± 0.8 (3.8–7.4)	1.7 ± 0.61 (0.6–2.5)
KN (μm)	10.55 ± 2.32 (5.44–14.86)	7.0 ± 1.4 (3.8–10.2)	10.7 ± 2.69 (7.8–15.0)
PN (μm)	11.54 ± 2.31 (6.20–15.69)	12.3 ± 1.5 (9.0–16.6)	12.4^#^
NA (μm)	11.81 ± 3.30 (6.10–20.25)	11.1 ± 1.2 (9.0–14.1)	7.8^#^
BL (μm)	23.36 ± 3.49 (12.30–30.90)	22.3 ± 1.9 (17.6–25.9)	21.2 ± 3.64 (15.6–24.9)
FF (μm)	15.73 ± 2.64 (8.20–20.87)	10.1 ± 1.3 (7.4–13.3)	14.3 ± 2.30 (9.2–18.2)
TL (μm)	39.09 ± 4.63 (28.70–47.09)	32.9 ± 2.2 (28.5–38.1)	35.5^#^
NL (μm)	1.61 ± 0.41 (1.04–2.95)	2.9 ± 0.4 (2.2–4.1)	–
NW (μm)	0.90 ± 0.20 (0.48–1.41)	1.3 ± 0.2 (1.0–1.6)	–
BW (μm)	1.80 ± 0.29 (1.13–2.33)	1.7 ± 0.2 (1.3–2.0)	2.3 ± 0.40 (1.6–3.1)
NI	1.07 ± 0.39 (0.46–1.86)	1.1 ± 0.2 (0.8–1.7)	1.7^#^
KI	1.10 ± 0.04 (1.05–1.18)	1.8 ± 0.3 (1.3–2.6)	1.3^#^
FI	0.69 ± 0.17 (0.40–1.36)	0.46 ± 0.02 (0.31–0.76)	0.69
Host	*Larimichthys crocea*	*Epinephelus fuscoguttatus*	*Carassius auratus* gibelio

BL, body length; BW, parasite maximum body width; FF, free flagellum length; FI (flagellar index) = FF/BL; KI (kinetoplast index) = PN/KN; KN, kinetoplast to mid-nucleus; TL, total length with flagellum; NA, mid-nucleus to anterior end; NI (nuclear index) = PN/NA; NL, nucleus length; NW, nucleus width; PK, posterior end to kinetoplast; PN, posterior end to mid-nucleus. ^#^Recalculated by Zhang *et al.* [48]

#### Molecular characterization and phylogenetic analysis

The total length of the amplified 18S rRNA sequences was 730 bp (accession number: PP897248–PP897259). The sequence from the 12 specimens was the same. Blast analysis of the newly obtained sequence indicated that it had the highest homology with *T. carassii* (98.4%). Homology with other identified *Trypanosoma* species infecting marine and freshwater fish in China was 92.3–96.9% and 97.3–98.4%, respectively.

**Figure 3:**
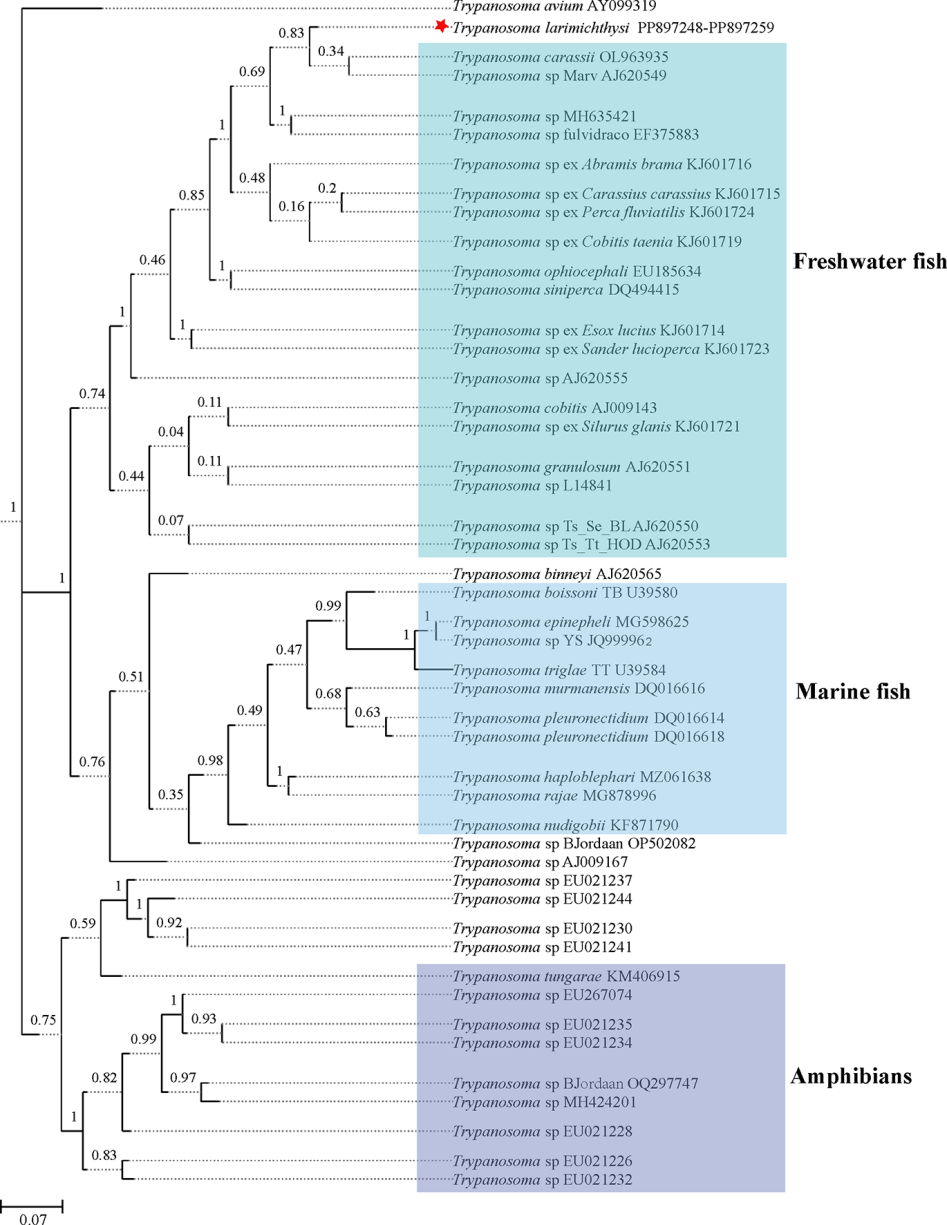
BI phylogenetic tree of trypanosomes based on 18S rDNA sequences. Red star representing the sequence obtained in the present study.

The results of both BI and ML phylogenetic trees constructed using the 18S partial fragment indicated that trypanosomes from the large yellow croaker (Fujian Province, China) clustered with freshwater fish trypanosomes (Fig. 3 and Supplementary file 3).

#### Condition factor and serum biochemistry

**Figure 4:**
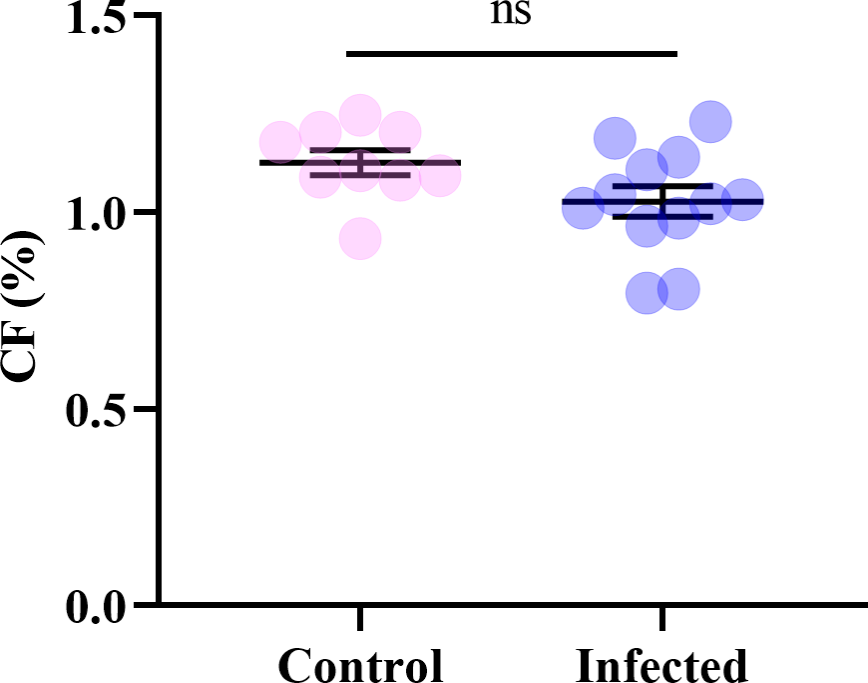
The condition factor of *L. crocea*.

**Figure 5:**
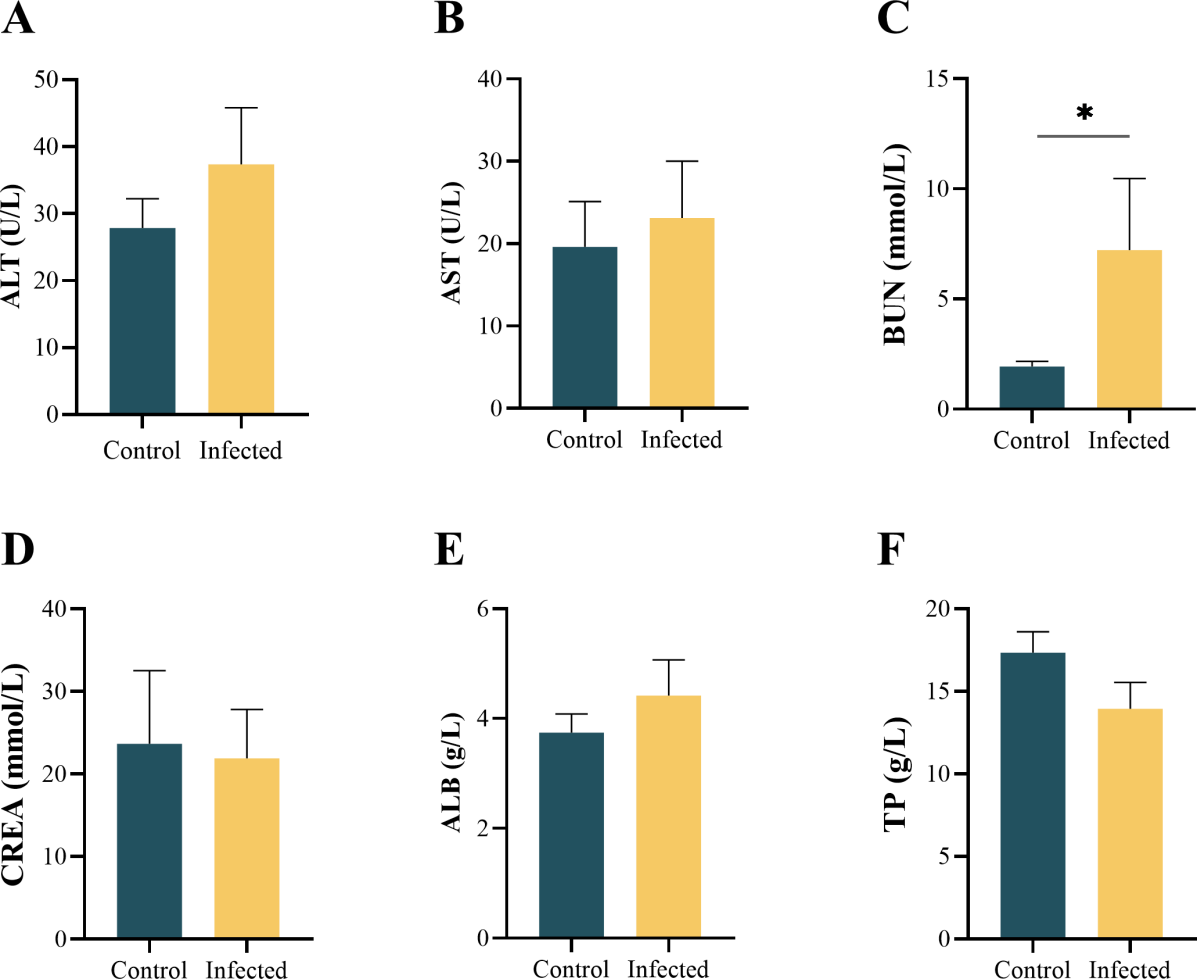
The blood biochemistry indices of *L. crocea*.

The condition factor of the Control group was slightly higher than that of the Infected group, but the difference between the two groups was not significant (*p* = 0.075 > 0.05, Fig. 4). The serum biochemical indices of large yellow croaker are shown in Figure 5 and Supplementary file 4. Trypanosomes infection significantly increased the concentration of BUN (*p* = 0.049 < 0.05). No significant changes were observed in the other indices, including ALT, AST, CREA, ALB and TP (*p* > 0.05 in all cases). The levels of ALT, AST and ALB were slightly elevated following trypanosome infection, accompanied by slight decreases in the levels of CREA and TP.

#### Histopathology

**Figure 6:**
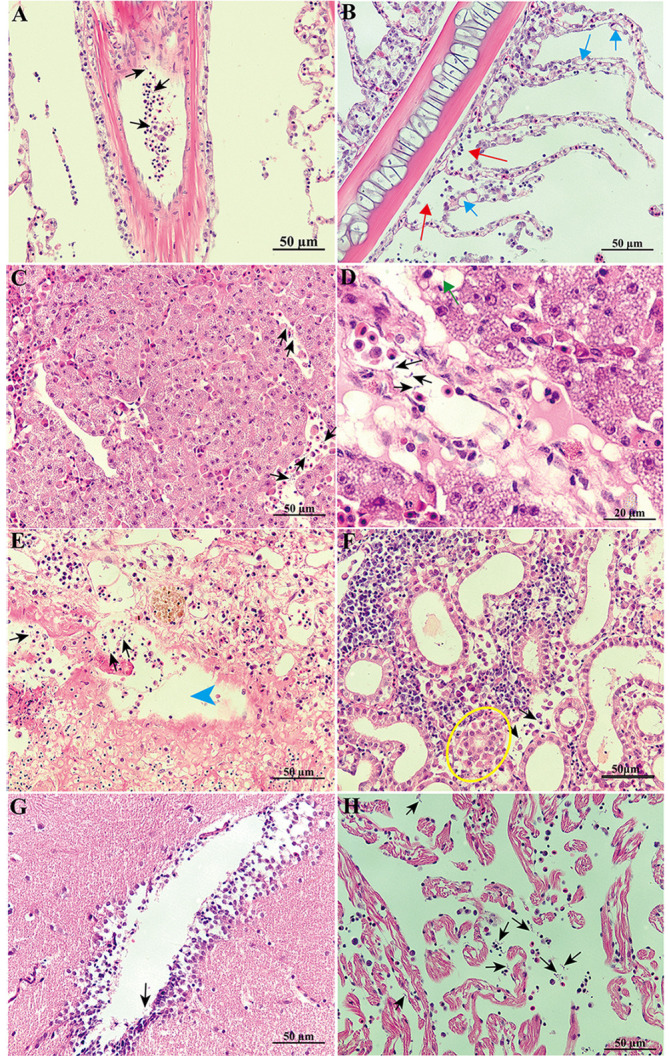
Histology of *L. crocea* infected with trypanosomes. A: trypanosomes (black arrow) were observed in vessels of the gill. B: hyperplasia (blue arrow) and gill epithelial detachment (red arrow) were evident in secondary lamellae. C: a massive amount of trypanosomes (black arrow) were inside the vessels of the liver. D: vacuolated hepatocytes (green arrow) were observed in the liver. E: trypanosomes (black arrow) were inside the spleen and atrophy of the white pulp was evident (blue arrowhead) in the areas near the location where the trypanosomes were located. F: the tubular epithelial cells in the kidney were detached (yellow cycle). Parasites were found in the brain (G) and heart (H). All scale bars = 50 μm (except in D, scale bar = 20 μm).

The histological observation indicated the presence of trypanosomes within all the analyzed tissues (Fig. 6). Trypanosomes were observed in the vessels of the gills (Fig. 6A). The integrity of the gill tissue was affected. Hyperplasia of epithelial cells was evident in the gill lamellae (Fig. 6B). Gill epithelial detachment and cellular debris from the gill tissue were also observed (Figs. 6A, 6b). Trypanosomes were presented in the hepatic vein (Figs. 6C, 6D). The liver tissue showed lipid accumulation (Fig. 6D), and indistinct demarcation of hepatocytes was also evident (Figs. 6C, 6D). A large number of trypomastigotes were also found in the spleen, and atrophy of the white pulp was evident in the areas near the location where the trypanosomes were located (Fig. 6E). Trypanosomes were located in the interlobular blood vessel of the kidney (Fig. 6F). Detachment of the tubular epithelial cells in the kidney was observed in close proximity to the parasites (Fig. 6F). H&E sections of heart and brain tissues also showed the presence of *Trypanosoma* (Figs. 6G, 6H).

#### Expression analysis of immune related genes

**Figure 7:**
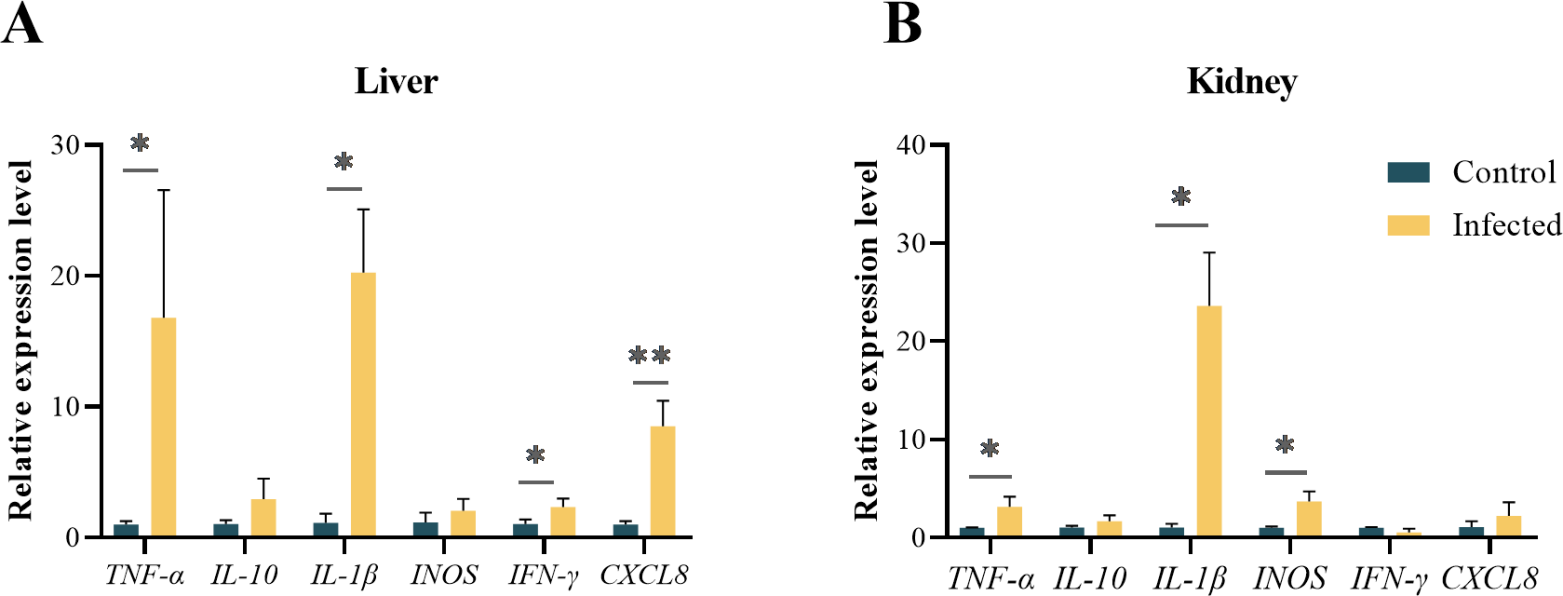
Expression levels of immune genes in the liver (A) and kidney (B) of large yellow croaker. “*”, 0.01 < *p* < 0.05. “**”, *p* < 0.01.

To understand the immune response elicited by trypanosomes infection in the large yellow croaker, the mRNA expression levels of immune-related genes, including *IFN-γ*, *TNF-α*, *IL-1β*, *CXCL8*, *IL-10* and *iNOS*, were measured in the liver and kidney (Fig. 7). Trypanosome infection significantly upregulated mRNA expression levels of *TNF-α*, *IL-1β*, *IFN-γ* and *CXCL8* (*p* < 0.05 in all cases) in the liver, with a trend toward increased expression of *IL-10* and *iNOS* (*p* > 0.05 in all cases) (Fig. 7A). In the kidney, trypanosome infection significantly increased mRNA expression levels of *TNF-α*, *IL-1β* and *iNOS* (*p* < 0.05 in all cases), with no alteration in the expression of *IL-10*, *IFN-γ* and *CXCL8* (*p* > 0.05 in all cases) (Fig. 7B).

## Discussion

Outbreaks of trypanosomiasis are not scarce in intensive aquaculture, especially in net-cages. It has been reported in various host species within the Order Perciformes, including Serranidae and Centropomidae [25, 40, 43], as well as in Cichliformes: Cichlidae [7], and Centrarchiformes: Centrarchidae [19]. In the present study, we document the first report of a trypanosomiasis outbreak in a new host species, *Larimichthys*, belonging to the Family Sciaenidae of the Order Perciformes.

### Identification of trypanosome species in large yellow croaker

Thirteen morphological parameters described by Gu *et al.* [14] were used as values and standards in morphological identification of *Trypanosoma*. The marine fish trypanosome, *Trypanosoma epinepheli* Su, Feng, Jiang, Guo, Liu & Xu, 2014 was first detected in the brown-marbled grouper (*Epinephelus fuscoguttatus*) [40], and subsequently, it was found in *Lates calcarifer* [25] and *Oreochromis niloticus* [4]. Comparative morphology showed significant differences between the *Trypanosoma* species in *L. crocea* and *T. epinepheli* (Table 1). Compared to *T. epinepheli*, the *Trypanosoma* species found in large yellow croaker possessed several distinct morphological features: a longer body length (39.1 *vs*. 32.9 μm), a greater KN distance (10.6 *vs*. 7.0 μm), an extended free flagellum length (15.7 *vs*. 10.1 μm), a larger flagellar index (FI: 0.69 *vs*. 0.46), a shorter PK distance (0.99 *vs*. 5.4 μm), a reduced nucleus length (1.6 *vs*. 2.9 μm), a narrower nucleus width (0.9 *vs*. 1.3 μm) and a smaller kinetoplast index (1.1 *vs*. 1.8). *Trypanosoma carassii* (Mitrofanov) Doflein, 1901, a common hemoflagellate parasite of freshwater fish, is known for its widest geographic and host range. The mensural values of KN, PN, BL, FF and FI were similar between the *Trypanosoma* species in *L. crocea* and *T*. *carassii* (Table 1). However, the two species differed in the following morphological characteristics: the *Trypanosoma* species in large yellow croaker has a more centrally located nucleus (NI: 1.07 *vs.* 1.7), a shorter PK distance (0.99 *vs.* 1.7 μm), a narrow body width (1.8 *vs.* 2.3 μm), a smaller kinetoplast index (1.1 *vs.* 1.3), a longer NA distance (11.8 *vs.* 7.8 μm) and a greater total length (39.09 *vs.* 35.5 μm).

The morphological plasticity of trypanosomes is high [4, 49], necessitating the use of molecular techniques for accurate species identification. The 18S rDNA has been widely used as a molecular marker for the identification of fish trypanosomes [21]. In the present study, partial fragments of the 18S rDNA gene were amplified, and all the sequences obtained from the large yellow croaker were identical, indicating a single *Trypanosoma* species infection at this site. The sequence showed 98.4% homology with *T. carassii*. Phylogenetic analysis also showed that the present trypanosome found in large yellow croaker clustered with *T. carassii* within a freshwater fish-infecting *Trypanosoma* clade, indicating a close phylogenetic relationship with *T. carassii*.

Fish trypanosomes were classified into two distinct clades, which corresponded to the trypanosomes found in freshwater and marine fish. Large yellow croaker is a marine fish. However, the trypanosome obtained from this marine fish was unexpectedly classified within the freshwater fish clade of *Trypanosoma*. This inconsistency is a novel finding in the taxonomy of fish trypanosomes. Although a distinct phylogenetic boundary exists between marine and freshwater fish trypanosomes, they may overlap in the estuarine ecosystem [49]. The water of stream “Jiaoxi” flows into Sanduao Bay, where the primary outbreak of trypanosomiasis occured in large yellow croaker. Thus, further study is needed to find the leech vector in the estuary. Additionally, the host specificity of trypanosomes is generally considered to be low, as evidenced by the ability of Nile tilapia to be experimentally infected with both marine and freshwater fish trypanosomes [4]. Thus, it is possible that the large yellow croaker could be infected with this trypanosome having a close relationship with the freshwater trypanosome clade.

Based on the differences in morphological characteristics, host species, and molecular data, the trypanosome from the large yellow croaker is regarded as a novel species of *Trypanosoma*, and we name it *T. larimichthysi* n. sp.

### The effect of Trypanosomiasis induced by *T. larimichthysi* on growth and blood biochemistry of large yellow croaker

Anemia induced by trypanosomes may alter the body conditions and somatic indices of the liver, spleen and heart in the host [44]. However, the growth of *L. crocea* in this study was not affected by *T. larimichthysi*, as indicated by condition factors. Similar results were observed in *Hypostomus* spp. and *Pterygoplichthys pardalis* [6, 39]. A possible explanation is that the time from illness to death is too short for a marked decrease in the body weight of large yellow croaker.

Blood biochemistry indices are an important indicator for evaluating the health status of fish host. Trypanosome infection has been reported to induce changes in the biochemical composition of infected fish [15, 19]. The indices, including TP, ALB, ALT, AST, BUN and CREA, are important indicators for evaluating liver and kidney function. In the present study, trypanosome infection significantly increased BUN levels, indicating kidney dysfunction. AST and ALT activities were higher in diseased fish, which was due to hepatocyte degeneration and changes in cell membrane permeability, leading to the escape of these substances [50]. The lower concentration of TP observed in infected fish is consistent with the results shown in *Hemibagrus macropterus* (syn. *Mystus macropterus*) infected by *T. hemibagri* [50]. A massive amount of proteins were used for wound repair, resulting in a decrease in serum TP content.

### The effect of Trypanosomiasis induced by *T. larimichthysi* on histology of large yellow croaker

Previous pathology studies have shown that fish trypanosome infection induced significant pathological changes in various tissues [7, 19, 25, 43, 50]. Trypanosome infection caused damage to the gills and proliferation of gill epithelial cells, and the presence of cell fragments. These obvious pathological changes in the gills would impact breathing and osmotic regulation in the large yellow croaker. The vacuolization and disorganized arrangement of hepatocytes indicated that the liver function of large yellow croaker was impaired. Atrophy of the white pulp in the spleen and detachment of renal tubular epithelial cells indicated pathological changes in these two tissues as well.

### The effect of Trypanosomiasis induced by *T. larimichthysi* on immune response of large yellow croaker

The modulation of innate immune responses in mammalian trypanosome infections has been studied extensively [3]. However, it is poorly understood in fish. Mammalian trypanosomes trigger a series of innate immune responses in the host via pathogen-associated molecular patterns, which activate macrophages to produce pro-inflammatory cytokines such as TNF-α, IFN-γ, IL-1 and NO [27, 34]. These Th1 cytokines are required for efficient control of the parasite burden in the early stages of infection and long-term host survival [26]. In the present study, *T. larimichthysi* infection significantly up-regulated the expression of pro-inflammatory genes encoding *TNF-α*, *IL-1β*, *CXCL8*, *IFN-γ* and *iNOS*, which is consistent with findings observed in goldfish infected with *T. carassii* [33]. These results indicate that the important role of Th1-like immune response in controlling *Trypanosoma* burden is conserved in vertebrates.

## Conclusions

In summary, a new species of trypanosomes named *T. larimichthysi* n. sp. was found in large yellow croaker by morphological and molecular identifications. The parasite infection did not affect the growth of *L. crocea*, but significantly increased the level of BUN. Pathological changes were observed in the gills, liver, spleen and kidney of the diseased fish infected with *T. larimichthysi*, and expression levels of pro-inflammatory genes, including *TNF-α*, *IL-1β*, *CXCL8*, *IFN-γ* and *iNOS*, were significantly up-regulated. These results indicate that the outbreak of trypanosomiasis induced significant negative effects on the health of large yellow croaker. Urgent research is needed on the biology, epidemiology, life history, prevention and control methods of this trypanosome.

## Figures and Tables

**Supplementary file 3:**
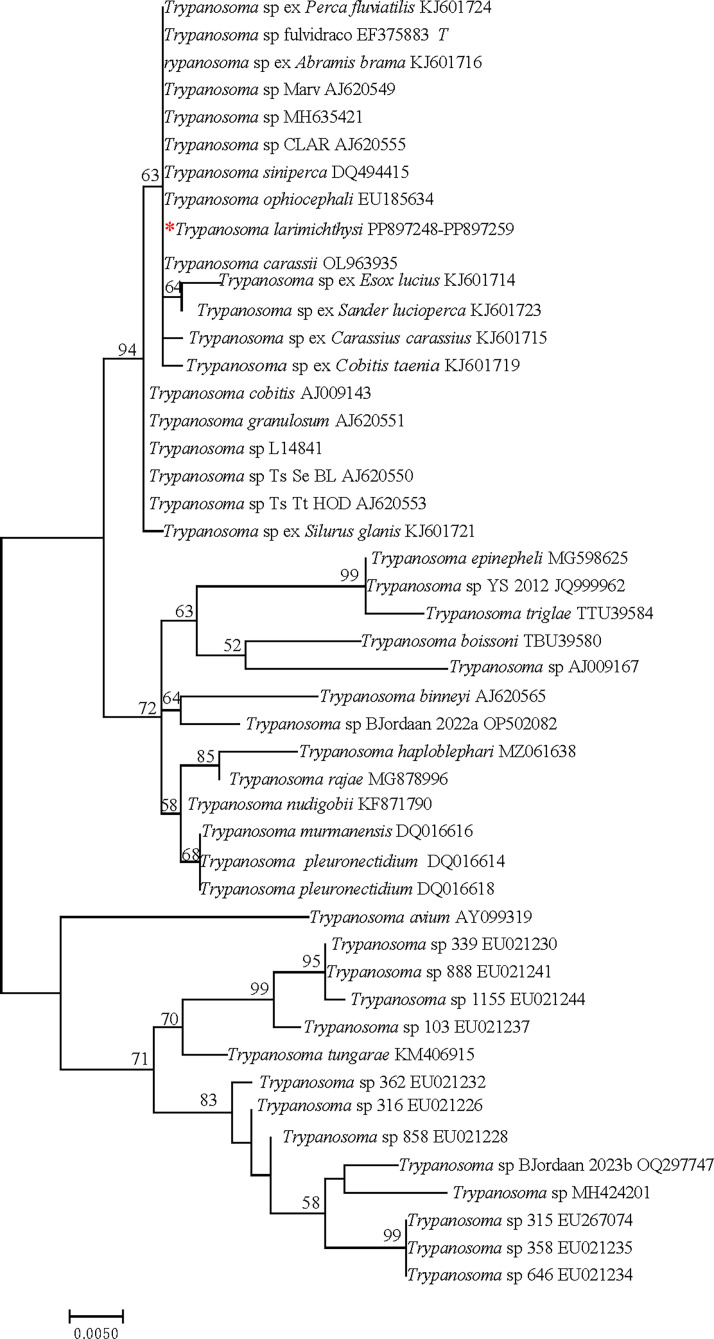
ML phylogenetic tree of trypanosomes based on 18S rDNA sequences. Red star represent the sequence obtained in the present study.

## Supplementary material

The supplementary material for this article can be found at https://www.parasite-journal.org/10.1051/parasite/2024078/olm. *Supplementary file 1*: Information on sampled large yellow croaker. *Supplementary file 2*: Primers used for qPCR.

*Supplementary file 4*: Serum biochemical parameters in large yellow croaker.
