# Supplementary material for: Identification of a new fish trypanosome from the large yellow croaker (Larimichthys crocea) and description of its impact on host pathology, blood biochemical parameters and immune responses
Source: Parasite. 2025 Jan 22;32:1. doi: 10.1051/parasite/2024078 (PMC11752739; doi:10.1051/parasite/2024078)
Supplement: Supplementary file 1 — Information on sampled large yellow croaker. [file parasite-32-1-s1.pdf]

Supplementary file 1. Information on sampled large yellow croaker. “+” indicates that the specimen was infected with trypanosomes, and uninfected samples are indicated by “-”.

Group	Sample ID	Length (cm)	Weigh (g)	Trypanosomes infection
Infected	1	20.6	103.9	+
	3	16.0	39.5	+
	4	19.5	73.0	+
	5	18.0	59.6	+
	6	21.5	122.3	+
	7	22.0	121.4	+
	10	20.2	65.5	+
	11	22.0	107.6	+
	13	18.7	67.5	+
	14	17.5	59.4	+
	15	21.8	83.4	+
	16	21.0	96.8	+
Control	2	16.0	38.2	-
	8	22.0	117.7	-
	9	26.0	211.5	-
	12	19.5	81.0	-
	17	20.5	93.8	-
	18	22.0	125.5	-
	19	24.5	183.4	-
	20	20.5	103.7	-
	21	18.0	63.1	-
