# Supplementary material for: Identification of a new fish trypanosome from the large yellow croaker (Larimichthys crocea) and description of its impact on host pathology, blood biochemical parameters and immune responses
Source: Parasite. 2025 Jan 22;32:1. doi: 10.1051/parasite/2024078 (PMC11752739; doi:10.1051/parasite/2024078)
Supplement: Supplementary file 2 — Primers used for qPCR. [file parasite-32-1-s2.pdf]

Supplementary file 2. Primers used for qPCR.

Gene	Primer sequence	Accession Number
β-Actin	Forward: 5′ – AGGGAAATCGTGCGTGACAT -3′	XM_027284923.1
	Reverse: 5′ – CCATACCGAGGAAGGATGGC -3′	
TNF-α	Forward: 5′ – GGGAAAACGCCTCACACCT -3′	NM_001303385.1
	Reverse: 5′- GGCGTTGTACCAACCCTGT -3′	
IL-10	Forward: 5′ – TGGGTGAGCTGAATCTGCTG -3′	XM_010738826.3
	Reverse: 5′ – CAACATCTGGCGACACTCAC -3′	
IL-1β	Forward: 5′ – ATCTGGCAAGGATCAGCTCA -3′	XM_010736551.3
	Reverse: 5′ – ACCAGTTGTTGTAGGGGACG -3′	
iNOS	Forward: 5′ – ATCCAGATGCCCTCCACTCT -3′	XM_019272008.2
	Reverse: 5′ – GAGGTGGATCTCTCCTGGGT -3′	
CXCL8	Forward: 5′ – CAGAATCTTCGTCGCCTCCA -3′	XM_010737667.3
	Reverse: 5′ – CCGATGGGTTTGCTCTCTGT -3′	
IFN-γ	Forward: 5′ – CAGACAGTCAGACAGGCGTT -3′	XM_010751697.3
	Reverse: 5′ – ACCACTGCCCTCACTATTGC -3′	
