# Supplementary material for: Identification of a new fish trypanosome from the large yellow croaker (Larimichthys crocea) and description of its impact on host pathology, blood biochemical parameters and immune responses
Source: Parasite. 2025 Jan 22;32:1. doi: 10.1051/parasite/2024078 (PMC11752739; doi:10.1051/parasite/2024078)
Supplement: Supplementary file 3 — Supplementary file 4: Serum biochemical parameters in large yellow croaker. [file parasite-32-1-s3.pdf]

Supplementary file 4. Serum biochemical parameters in large yellow croaker.

**Group**	**ALT (U/L)**	**AST (U/L)**	**BUN (mg/dL)**	**CREA (μmol/L)**	**TP (g/L)**	**ALB (g/L)**
Control	23.15	25.84	2.18	17.34	18.41	4.08
	31.78	17.69	1.70	19.82	15.95	3.41
	28.62	15.35	1.92	33.78	17.68	3.74
Infected	26.88	24.95	10.73	28.65	13.33	4.99
	54.08	28.91	6.63	19.46	11.55	3.12
	31.09	15.47	4.30	17.57	16.97	5.14
